# Episodic confusional state: Due to insulinoma

**DOI:** 10.4103/0019-5545.43636

**Published:** 2008

**Authors:** Venkatesan Jagadheesan, Stelina Sophie Dina Suresh

**Affiliations:** Department of Psychiatry, Thanjavur Medical College, Thanjavur, India

**Keywords:** Confusional state, insulinoma, psychosis

## Abstract

This case report deals with 45-year-old male who came for consultation in the psychiatry department for the persisting symptoms, after consulting various departments with no relief. He had episodes of confusion with disorganized behavior, restlessness, and symptoms like talking irrelevantly once a week lasting up to 10-30 min in the preceding six months. Investigations like computerized tomography scan, electroencephalogram were not contributory. While under observation in our ward for evaluation and diagnosis, one such episode with intense sweating and clouding of consciousness was witnessed and helped in clinching the diagnosis of insulinoma. The case is reported for its rarity and as one of the causes of episodic confusional state.

## INTRODUCTION

Insulinoma is a neuroendocrine tumour of pancreas. It is also the most common cause of hyperinsulinemic hypoglycemia. The incidence is 1 to 4 per million.[1] They invariably present with signs and symptoms related to hypoglycemia[2], viz:

- Psychiatric manifestations
- Signs of autonomic disturbance
- Chronic sequlae

The disorders which they produce are extremely varied and intermittent with normal health between the episodes. Recurrent confusional state is typical of insulinoma.[3] Most of the patients present with neuropsychiatric symptoms and are often misdiagnosed as dissociative disorder[4] or psychosis.[1,5] The diagnosis of the condition is often delayed resulting in prolonged ill health.

## CASE REPORT

A 45-year-old male presented with complaints of episodic dizziness occurring two – three times per day in the past eight months. He had attended Neurology out patient department (OPD) in October 2006 for episodes of giddiness. The Computerized Tomography (CT) Scan taken at the time was normal and no neurological disorder was detected at that time. He came for psychiatric consultation eight months later on his own. Meanwhile, he had been treated symptomatically at general medical and surgical departments. Random blood sugar showed 58 mg% at the time of admission.

During the onset of the illness, he was having only episodes of giddiness lasting 1-2 min. This progressed to episodes of confusion with other behavioral changes lasting 5-30 min. During the episodes of confusion, patient had slurred speech, altered behavior like banging his head against the wall, walking aimlessly and talking irrelevantly. Patient was having intense sweating and autonomic symptoms during the episodes. Clonic movements and tremors of both arms were also observed during some episodes.

During the episodes he was not oriented to time, place and person. He was also completely amnesic for the episodes after he came out of the episodes. Episodes initially occurred in the afternoon and then more in the nights during sleep and in the evenings. He had no incontinence of urine during the episodes. He never had any generalized seizures or aura or premonitory symptoms preceding the episodes.

General and systemic examination was normal. Central nervous system examination showed mild dysarthria. Psychomotor activity was normal and mood was euthymic. Thought and perception were normal. Cognitive functions showed mild impairment. Insight was present. At psychiatric OPD he was diagnosed as episodic confusional state and admitted for observation and to exclude insulinoma.

**Figure 1:**
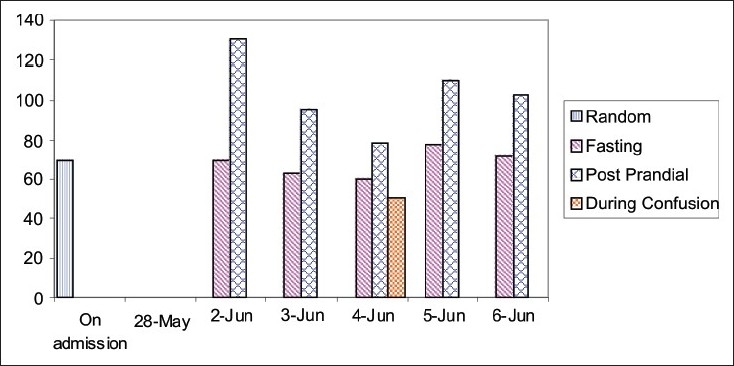
Graph showing blood sugar levels 50 mg% during one such episode of confusion

After admission in the ward, he developed an episode which was reversed by administration of glucose. Patient was in confusional state with autonomic symptoms like tachycardia and profuse sweating. Clonic movements of limbs were also observed. The patient was not oriented to time, place and person. Blood glucose level showed 50 mg% during this episode [Figure 1].

## INVESTIGATIONS

**Figure 2:**
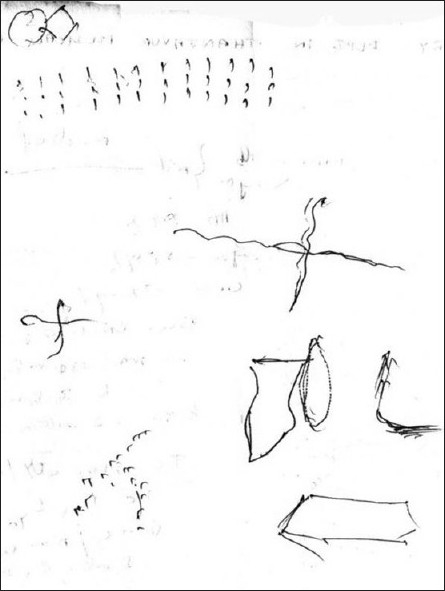
Bender gestalt test

Random blood sugar at the time of admission showed 70 mg%. Serum Plasma insulin was 2.36 IU/ml (normal <6µU/ml) and C peptide 0.19 ng/ml (normal < 2ng/ml) during one episode. Other blood tests were normal. CT scan brain done in October 2006 and June 2007 were normal. Abdominal ultra sonogram and CT scan abdomen done was normal. EEG recorded 15 min after an episode was normal. Serum prolactin levels taken 15 min after an episode was normal (100 IU). Routine Magnetic Resonance Imaging (MRI) abdomen could not locate any tumour. Psychological tests administered revealed mild cognitive impairment in-between the episodes. Bender Gestalt test (BGT) [Figure 2] showed rotation, distortion and separation. There was impairment in visuospatial perception. MMSE score was 20. In Wechsler Memory Scale he was having M.Q. of 85. In WAIS he had I.Q. of 85.

## DISCUSSION

Misdiagnosis of insulinoma is common and to make a diagnosis of insulinoma, the physician must consider it. Hypoglycemia can mimic epileptic seizures and can manifest with seizures.[7] Complex partial seizure was ruled out in our patient by:

- Observation of autonomic features and total behavior during the episodes.
- No aura or postictal confusion observed.
- A course of carbamazepine 200 mg, 1 tds and eptoin, 2 hs, started after admission and continued for ten days, did not control the attacks.
- Electroencephalogram (EEG) showed no abnormal findings after an attack.
- Prolactin levels from a sample taken 15 min after an attack was normal.

It is worth mentioning that in any patient with refractory seizures we have to think of metabolic cause for its aetiology.[8,9] Insulinoma can also present with normoglycemia even after fasting.[10] Blood samples were taken after overnight fasting for three consecutive days in our patient. A normal test should be interpreted in the light of clinical symptoms.[11] Nekamura *et al*.[4] reported a case of insulinoma presenting as hysteria. Case reported by Pandey *et al*.[1] presented with dizziness and altered behavior[2] and irrelevant talk. Insulinoma presents with various neuroglycopenic manifestations which get reversed by administration of glucose. Recurrent attacks of hypoglycemia can cause some residual neurological deficits.[3,12–14] Impairment of cognitive functions was present in our patient and he was having mild persistent dysarthria. Marks[15] has categorized the symptoms occurring in insulinoma into four categories:

- Acute neuroglycopenia
- Sub acute neuroglycopenia
- Chronic neuroglycopenia
- Hyperinsulinemic neuropathy.

Insulinomas are diagnosed by[16] (a) low blood glucose levels 40-50mg%, (b) symptoms of hypoglycemia and (c) dramatic reversal of central nervous system (CNS) abnormalities by glucose administration (Whipple's triad). Supervised fasting for 48-72 hrs can be conducted to confirm the diagnosis. Plasma insulin, C-peptide levels and plasma insulin/glucose ratio are diagnostic.[7] Insulinomas are usually less than 2 cms in 80% of the cases. Localization of insulinomas prior to surgery is a topic of debate.[17] Imaging techniques such as computed tomography, MRI and ultrasonography lack sensitivity. Imaging procedures such as selective pancreatic arteriography with calcium stimulation can help in localization of the tumour.[18] Identification at the time of surgery with aid of intra operative ultrasonography (IOUS) can be done.[18,19] Surgery is the most effective therapy for insulinoma.[20] Medical therapy is limited to diazoxide, calcium channel blockers, dilantin and somatostatin. The patient was followed up for next two months and he continued with the same symptoms. He was referred to higher centres for further management.

## CONCLUSION

Insulinoma should be thought of as a cause for episodic confusional state. Insulinoma can be mistaken as complex partial seizure since it closely mimics it. Insulinomas can be present with neuropsychiatric symptoms creating a diagnostic dilemma. Misdiagnosis and delayed diagnosis of insulinoma is common[6] and it can lead to ill health and permanent sequlae. So, to make a diagnosis of insulinoma one must consider it.
